# Gene Expression Profiling and Qualitative Characteristics in Delaying Flesh Softening of Avocado Fruits

**DOI:** 10.3390/genes15070860

**Published:** 2024-07-01

**Authors:** Ourania Anagnostopoulou, Georgios Tsaniklidis, Konstantinos Paschalidis, Filippos Ververidis

**Affiliations:** 1 Department of Agriculture, School of Agricultural Sciences, Hellenic Mediterranean University (HMU), 71410 Heraklion, Greece; raniaanagn@gmail.com (O.A.); ververidis@hmu.gr (F.V.); 2 Hellenic Agricultural Organization (ELGO-DIMITRA), Institute of Olive Tree, Subtropical Crops and Viticulture, 73134 Chania, Greece; tsaniklidis@elgo.iosv.gr

**Keywords:** avocado, qPCR, PAL, β-gal, beta-galactosidase, APX, plastic film, storage, quality, Ethrel

## Abstract

In this research, qualitative characteristics were studied under different post-harvest treatments in Hass and Fuerte cultivars of avocado (*Persea americana*) fruits. The post-harvest treatments performed in fruits of these cultivars comprised Ethrel application and plastic film (membrane) covering. The measurements of qualitative characteristics were related to color; flesh consistency; measurements of titratable acidity, total soluble solids, percentage of total phenolic contents, and ascorbic peroxidase activity; and the real-time (quantitative) polymerase chain reaction (qPCR) of gene expression and enzyme activities of phenylalanine ammonia-lyase (PAL) and beta-galactosidase (β-gal). The experiments found that the application of plastic film has excellent results in retaining qualitative characteristics and enzyme activities via maintaining firmness in higher levels. The plastic film covering appeared to delay ripening without the use of chemicals and, therefore, it has the potential to extend the duration of the post-harvest life of the avocado fruit. Variations between the two cultivars were found in the measurements of total soluble solids (Fuerte cultivar showed an increase of 22%, whereas Hass cultivar showed an increase of 120% in Brix values) and total phenolic contents (Fuerte cultivar showed a decrease of 16% and Hass cultivar showed an increase of 29%). It is worth noting that PAL’s activity increased significantly (over 44%), as compared to other treatments, and β-galactosidase’s activity decreased, as compared to other treatments. In conclusion, plastic film covering results in a decrease in the activity of β-galactosidase, as shown by the reaction of hydrolysis (enzyme activity) but also from the expression of the related genes.

## 1. Introduction

The avocado (*P. americana*) is a fruit with a history dating back approximately 10,000 years. It is produced from tropical trees and is characterized by its pear shape and blackish-green color. The fruit has high nutritional value, a creamy texture, and a unique taste. Due to its commercial importance, the avocado is often referred to as “Green Gold”. The global avocado market (gross production value was about 5.812 billion USD in 2018) has been experiencing a steady increase in value and is expected to continue to do so in the future [1].

Avocados are highly nutritious fruits due to their significant content of essential unsaturated fatty acids, vitamins, potassium, and fiber, while also exhibiting a low total soluble solids content. So, they are frequently included in high-nutrient diets, helping in reducing the risk of metabolic syndrome, which is a combination of metabolic disorders, such as obesity, diabetes mellitus, and hypertension that are risk factors for cardiovascular disease, in humans [2]. Avocado fruits contain over 20 vitamins and minerals, including potassium (which helps regulate blood pressure), lutein (which helps in eye health), and folate (which is vital for cell health). Avocados are also a good source of B, A, and C vitamins, as well as vitamins D and E, which are involved in the metabolism of carbohydrates, fats, and proteins [3]. Clinical studies have shown that avocado consumption helps in heart health, circulation, and normal blood pressure levels [2].

The texture of fruits and vegetables is determined by various factors, including the genotype of each cultivar, the degree of ripeness, and water content. Softening of the fruit occurs due to metabolic changes in the cell walls of the tissues and a loss of cell turgidity, which ultimately leads to a decline in quality [4]. To summarize, food quality is assessed firstly by appearance, which includes color, gloss, and size, and secondly by texture, total soluble solids (TSS), and titratable acidity (TA) [5]. These parameters have the ability to provide important information to both researchers and consumers when selecting foods.

Packages should be constructed from paper boxes or, alternatively, covered with paper to ensure softness and prevent scratching of the fruit’s skin. They should be placed in the shade and left in the field for the shortest possible time before being transported immediately to the collection and sale location or placed in refrigerators [6]. The optimum storage temperature for most avocado cultivars is 7.2 to 7.5 °C, except for West Indian cultivars which should be stored at 12 °C. Most cultivars can be stored for about 4 weeks at a relative humidity of 85–90% during storage. Plastic film is commonly used to extend the shelf life of various fruits and vegetables (e.g., cucumbers, pears). By wrapping each fruit individually, the fruit is allowed to continue its physiological functions, reduce its weight loss, extend its shelf life, and improve its quality. Usually, fruits that are intended to be wrapped in plastic film must be treated with a fungicide after harvesting. If this treatment is not carried out, a favorable micro-environment for fungal growth is created between the skin and the plastic film. The procedure of covering the fruit with plastic film for prolonged preservation is usually carried out on each fruit individually, although modified-atmosphere packaging is also available that wraps a small number of fruits. Before covering the fruit with plastic film, it is recommended that gibberellin is applied to slow down the aging of the skin.

The result of the application of individual packaging with plastic film is the creation of a saturated microenvironment around the perimeter of the fruit. This has the effect of preserving it for a prolonged period of time, as a consequence of the reduction in the respiratory process.

Ethylene is a plant hormone that regulates a multitude of physiological processes during plant growth. It is one of the most widely used plant hormones in agriculture. Ethylene, in its gaseous form, is very difficult to apply in the field due to its high diffusion rate. For this reason, the substance ethephon (2-chloroethylphosphonic acid) is used instead. Ethephon is a plant growth regulator, and upon metabolization by the plant, it is converted into ethylene. The trade name of the formulation that was used is Ethrel [7,8].

Phenylalanine ammonia-lyase (PAL) is an enzyme that plays an important role in the metabolism of phenolic compounds. The activity and expression of the PAL gene are regulated by a cultivar of biotic and abiotic factors, such as hormones, nutrient levels, light (through its action on phytochrome), fungal infection, injury, and other stresses [9,10]. The effect of any or all of the above factors causes replication of the messenger RNA encoding the PAL protein, resulting in an increase in PAL synthesis, which results in the synthesis of phenolic components [10].

β-galactosidase (β-GAL) is implicated in several physiological processes and plays a pivotal role in seed germination, cell growth, and fruit ripening, where extensive cell wall loosening takes place [11]. β-galactosidase affects the softening of flesh tissue, which means the maturation and tightening of tissue molecules. During ripening, fruit softening involves a series of programmed changes in the polysaccharides of the primary cell wall and intercellular space, which results in weakening. During these changes, the polysaccharides are degraded and the bonds that bind them are changed, resulting in the swelling of the wall components and a reduction in the adhesion between them. It is usually accompanied by a decrease in the contact surface of the cells and an increase in the air areas between the cells [11].

Targeted genome technologies are a young field in the metabolic engineering of plants. We have previously established single-gene-based variations in metabolite composition directly breeding for specific fruit nutritional and/or anti-nutritional needs. Real-time (quantitative) polymerase chain reaction (qPCR) has also been extensively used in molecular diagnostics owing to its enormous sensitivity and specificity. Our previous findings highlight transcription patterns in a plethora of genes related to ripening and to biotic/abiotic stress cross-tolerance [12], and the dual role of β-GAL was revealed not only in maturation, but also in the metabolism during post-harvest homeostasis and cold acclimation [11]. The present data establish a relationship between gene expression levels and physiological–metabolic phenotypes of avocado fruit. The effect of Ethrel was examined in accelerating uniform ripening, and the effect of plastic film covering on ripening delay without the use of chemicals was examined in two avocado cultivars, in an effort to obtain ripening uniformity and extension of the post-harvest life duration in avocado fruit. In this effort, measurements of qualitative characteristics were performed regarding color, flesh consistency, titratable acidity, total soluble solids, total phenolic contents, ascorbic peroxidase activity, and qPCR gene expression experiments and the activities of PAL and β-gal.

## 2. Materials and Methods

### 2.1. Plant Material and Treatments

For this experiment, 75 avocados from two different cultivars, Hass and Fuerte, were used. These cultivars were selected as they represent the most preferable/commercial Cretan avocado cultivars intended for European exportation. To ensure a reserve of fruit in case of problems, more than 90 fruits per cultivar were obtained. The fruits were harvested the same day and had similar size. The avocados were supplied by Synergatiki S.A. from Chania, Crete, under the trade name “Creta Star”. The fruits were grouped together and divided into three groups. For each cultivar, two groups of fruits were treated, while a third group served as a control with no treatment applied. Each group comprised 25 fruits.

At the beginning of the experiment, the first treatment was spraying with Ethrel (2-chloroethylphosphonic acid) with a concentration of 250 μL per 0.5 L on 25 avocado fruits of Hass and Fuerte cultivars, respectively. More specifically, the fruits to be sprayed were left at room temperature for about 12 h to be more susceptible to the Ethrel treatment [13]. The avocados were transferred to a greenhouse and placed on a clean workbench with a spacing between them to ensure better and more accurate coverage with Ethrel. The fruit was then sprayed evenly around the perimeter with Ethrel. Once the process was complete, they were transferred to 20 L plastic chambers in the greenhouse and hermetically sealed. Subsequently, they were moved to the preservation chamber and kept at 10 °C for 21 days.

The second treatment performed for this experiment was the application of plastic film (membrane). Each fruit was independently wrapped with commercial plastic film (PVC material, thickness 10 micron). The wrapping of the film on the fruits was performed accurately and methodically on each fruit out of the 25 that were separated from the group for this treatment. The first step for successful results was to carefully clean each fruit with a soft absorbent paper so that it was completely dry with no trace of moisture on its surface. Square pieces of plastic film were then cut out and each fruit was very carefully and tightly covered with the plastic film in such a way that there was as little air enclosed as possible, or none. For the best coverage of the fruit, two layers of plastic film were applied to each fruit. This procedure was performed because any moisture trapped within the film over time would have a negative effect on fruit preservation, for example, the formation of rot and eventual softening of the tissue in the infected areas. After wrapping, the fruits were then transferred to the preservation chamber for a period of 21 days.

After the period of time the fruit had been in the preservation chamber, fruit samples from the three treatments (which had been in the preservation chamber for the last 21 days) were stored in a hyperfreezer at −80 °C. The storage procedure for the samples was as follows: Five fruits were selected for each of the six measurement categories (untreated, plastic film treatment, and Ethrel treatment for Hass and Fuerte). These fruits were cut lengthwise to remove the seed, and the flesh samples were then cut into cubes. These samples were selected so that they were away from the seed and the skin. 

Subsequently, these samples from the five fruits were mixed and stored in 50 mL falcon tubes in a hyperfreezer at −80 °C. Some of these experiments were carried out at the Institute of Viticulture of Heraklion in the Hellenic Agricultural Organization-Demeter. On the days of the experiments, the samples were transported from the hyperfreezer located at the laboratory site to the IALAI site. During the transfer, a small portable refrigerator for transporting drugs was used to avoid defrosting of the samples.

### 2.2. Color Measurement Process with Colorimeter

The color measurement process was conducted using a Konica Minolta CR-300 Colorimeter machine (manufactured in Japan, Tokyo by Konica Minolta) on eight avocado fruits of Hass and Fuerte cultivars, respectively. More specifically, measurements were taken on each avocado fruit at four points along the outside of the fruit, with two coordinates (L-a-b) and (L-C-h). They were then transferred to the preservation chamber for 21 days at 10 °C. After the period of time the avocados were in the preservation chamber, the procedure was repeated on 8 fruits from each treatment (24 fruits in total).

### 2.3. Procedure for Measuring Titratable Acidity

For the measurement of titratable acidity, 5 g of frozen avocado tissue was taken for each of the eight measurement categories (control, 21 days untreated, plastic film treatment, and Ethrel treatment for both cultivars). The tissue was first pulverized in a Multi multi-cutter and then transferred to the mortar to create a relatively uniform paste. Meanwhile, the samples were transferred to 50 mL centrifuge tubes (falcons) with the addition of 30 mL of deionized water. The samples (three replications) were then stirred for a few minutes in a vortex. Then, for homogenization, they were centrifuged at 8000 rpm for 10 min and, after 10 min, the centrifuge procedure was repeated at 10,000 rpm for 3 min. Then, a rapid filtration funnel with a filter paper was placed in a volumetric cylinder to separate the solid parts from the liquids, as a result of which a clear supernatant was obtained. The supernatants were then transferred to calibrated 50 mL flasks, and the volumes were adjusted to 50 mL with deionized water. The titratable acidity was determined by titrating 15 mL of aqueous avocado extracts with 0.01 M NaOH, using phenolphthalein (1%) as an indicator. Deionized water was the blank control. Measurements were performed in triplicates for each addition. The results are expressed as the content in mL of NaOH (0.01 M) consumed until an indication of permanent color change occurred. An amount of 1 mL of 0.01 M NaOH equals 0.01 milliequivalents of Na.

### 2.4. Total Soluble Solids

The estimation of total soluble solids is performed by using the Optica HR-130 portable refractometer (manufactured in Italy, Ponteranica (BG) by OPTIKA S.R.L.). The refractometer measures the refractive index in a liquid and gives a value corresponding to the Brix scale. This means that total soluble solids are calculated as a percentage of Brix degrees (% Brix), where 1 Brix degree is equivalent to 1 g of sucrose per 100 g of aqueous solution. An increase in Brix points indicates that the higher the Brix value on the scale, the higher the total soluble solid content appears to be.

The fruit samples (three repetitions) that were used were 5 gr of tissue from 3 different fruits of each measurement category. The ratio of tissue weight with the addition of deionized water was 1/1. Stirring was then performed in the Vortex for 1 min for each sample. The samples were then centrifuged for 10 min at 9000 rpm. Finally, after the 10 min had passed, the centrifuge result was checked so that a quantity of deionized water was added, and centrifugation was repeated for another 5 min. The measurements of total soluble solids were carried out at 20 °C. The same methodology was also followed for the pH measurement.

### 2.5. Determination of Flesh Consistency

Measuring the variation in the hardness was performed by using the Chatillon model DPP-10 (manufactured in United States, Florida by Chatillon) penetrometer with an 8 mm piston [11]. The resistance of the flesh to pressure was assessed by inserting the piston of a certain diameter to a certain depth. The resistance to pressure is expressed in Kg/cm^2^ and the diameter of the piston used should always be indicated. An amount of 1 kg/cm^2^ (kilograms force per square centimeter) equals 9.806614 N/cm^2^ (newtons per square centimeter). Measurements were made on ten random fruits from each cultivar (Hass and Fuerte) for all measurement categories.

### 2.6. Determination of Respiration

In the ripening of climacteric fruits, respiration plays an important role in their post-harvest life. The beginning of the climacteric period also marks the end of the storage life of the product as irreversible aging begins, leading to commercial maturity, overripening, and quality deterioration [11]. For the determination of respiration, 16 1000 mL plastic containers were used for each of the treatments for both cultivars. One avocado was placed in each container. Avocados were kept closed in the containers for 36 h. After this time, the CO_2_ content within the containers was measured using the PBI Dansensor Checkpoint (manufacturer Ametek Mocon) machine. With the help of the measuring mouthpiece and a needle, a hole was drilled in the top of the containers and then the CO_2_ content % was calculated and is expressed as respiration rate in mL CO_2_ kg^−1^ h^−1^.

### 2.7. PAL Assay

The extraction of the crude enzyme was performed with 4 mL of an extraction buffer (0.1 M potassium phosphate, pH 7.8) with 0.05 g of PVP for 1 g of tissue. PAL activity was assayed following the method of [14], with some modifications. One millimeter of crude enzyme extraction solution was incubated with 4 mL of borate buffer (50 mmol/lit, pH 8.8) and 1 mL of L-phenylalanine (20 mmol/lit), for 60 min, at 37 °C. The reaction was terminated by adding 0.2 mL of HCl 6 M. The activity of PAL was determined by the production of cinnamate, which was measured by the absorbance change at 290 nm. The blank was the crude enzyme preparation mixed with L-phenylalanine, with zero-time incubation. The specific activity is expressed as units where one unit is defined as increase of one unit of absorption/min* 1 mg protein. The PAL assay was performed in triplicate for all samples [14].

### 2.8. Total Phenolics Content

The total phenolics concentration was determined with the Folin–Ciocalteu reagent and according to [15] and measured in three repetitions. Briefly, in a tube, a mixture of 0.2 mL of Folin–Ciocalteu reagent, 2.6 mL of deionized water, and 0.2 mL of diluted extract was vortexed and then was allowed to stand at 20 °C for 6 min under darkness. Afterwards, 2 mL of Na_2_CO_3_ (7%, *w*/*v*) was added and the tube was vortexed and incubated at 20 °C for 90 min. Absorbance was measured at 750 nm with a spectrophotometer (Heλios Gamma 7 Delta, Spectronic Unicam, UK) versus a blank. The results are expressed as gallic acid equivalents (GAE) on a fresh weight basis and according to the corresponding calibration curves.

### 2.9. APX Activity Assay

Total APX activity was assessed using the method proposed by [16] with slight modifications and measured in three repetitions. A tissue sample of 400 mg was frozen in liquid nitrogen; ground; mixed with 2 mL of extraction buffer containing 50 mM Na-phosphate (pH 7.0), 0.25 mM EDTA (Sigmae-Aldrich, St. Louis, MI, USA), 2% soluble polyvinylpyrrolidone-25 PVP (MP Biochemicals, Eschwege, Germany), and 4 mM L-AsA; and centrifuged at 13,000× *g* for 10 min at 2 °C. The supernatant was collected, filtered, and immediately used for the estimation of APX activity with a reaction solution containing 25 mM Na-phosphate (pH 7.0), 0.1 mM EDTA, 0.2 mM H_2_O_2_, and 4 mM L-AsA. A protein extract of 200 mL with 850 mL of the reaction solution was used to perform the assay. The oxidation rate of L-AsA was photometrically assayed at 265 nm using the Shimadzu (Tokyo, Japan) UV-1700 spectrophotometer and UV cuvettes (Ratiolab, Dreieich, Germany) in 10 min intervals at room temperature. Predefined concentrations of AsA were used for the reference curve.

### 2.10. β-Gal Activity Assay

The β-Gal assay was performed using the method described by [17] with some modifications regarding the enzyme substrate to make it suitable for the β-Gal assay and was measured in three repetitions. The reaction mixture contained 200 mL of McInvaine solution (0.2 mΜ Na_2_HPO_4_ with pH adjusted at 4.6 with 0.1 M citric acid) and 200 mL of 3% *w*/*v* 4-nitrophenyl β-D-galactopyranoside (Sigma-Aldirch, St. Louis, MI, USA), as the reaction extract containing about 30 μg of total protein was used. The reaction mixture was incubated at 30 °C for 30 min. Adding 1 mL of 0.5 M Na_2_CO_3_, pH 10.5, terminated the reaction. The production of nitrophenol was assessed spectrophotometrically at 410 nM using a UV-1700 photometer (Shimadzu, Tokyo, Japan).

### 2.11. qPCR Experiments

Total RNAs were extracted from bark tissue using the method described in [18]. Briefly, plant tissue was ground to 1/10 *w*/*v* in “lysis buffer” (8 M GuHCl, 25 mM EDTA, 1% Sarcosyl, 2% Triton X-100, 25 mM sodium citrate, 0.2 M sodium acetate, pH adjusted to 5.2 with acetic acid). The lysate was incubated at 65 °C for 10 min and then centrifuged at 16,000× *g* for 10 min. An amount of 500 μL of the supernatant was transferred to a new tube and 625 μL of absolute ethanol was added (to obtain a 55.5% final concentration). Then, the mixture flowed through a silica column (FT-2.0 Filter-Tube Spin-ColumnSystem, G. Kisker GbR, Steinfurt, Germany) by centrifugation at 1500× *g* for 10 min. The column was washed once with 700 μL of “wash buffer 1” (4 M GuHCl, 25 mM Tris–HCl pH 6.6, and 60% ethanol) and twice (700 and 400 μL, respectively) with “wash buffer2” (2 mM Tris–HCl pH 7.0, 20 mM NaCl, and 80% ethanol) by centrifugation at 8000× *g* for 1 min. RNA was finally recovered in 50 μL of preheated (80 °C) nuclease-free elution buffer (10 mM Tris–HCl, pH 8.0).

Subsequently, the total RNA was treated with DNAase I (ThermoFisher, Waltham, MA, USA), and the inactivation of DNAse was performed with the application of heat (74 °C). cDNA synthesis was performed with the Superscript II cDNA synthesis kit (ThermoFisher, Waltham, MA, USA) according to the manufacturer’s instructions. The resulting first-strand cDNA was normalized for the expression of the housekeeping gene of Actin. qPCR experiments were performed with the use of the PowerUp™ SYBR^®^ Green Master Mix (ThermoFisher, Waltham, MA, USA) with a QuantStudio 3 Real-Time PCR System (ThermoFisher, Waltham, MA, USA). The relative quantification of gene expression was performed as previously described [19]. For all samples, qPCR reactions were performed in triplicate.

### 2.12. Statistical Analyses

Analysis of variance (ANOVA) was performed by using SPSS 28 software (SPSS, Chicago, IL, USA) and was followed by pairwise post hoc analyses (Student–Newman–Keuls test) to determine which means differed significantly at *p* ≤ 5%.

## 3. Results

### 3.1. Color Measurement Results

**Figure 1:**
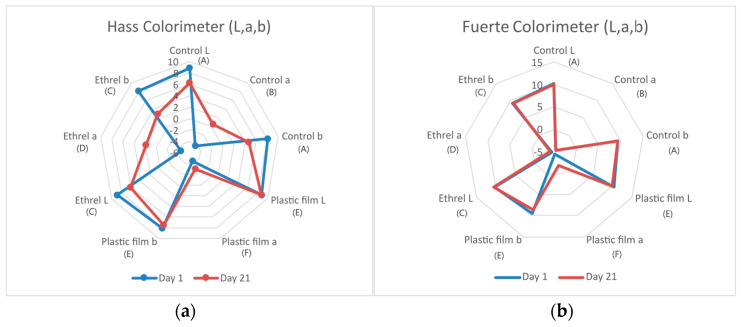
Colorimeter results of Hass. In the plastic film treatment, there was a small change of the color. The Ethrel treatment and the control have a significant color change (**a**). In the Fuerte cultivar, the fruit color was very close to the day 1 color and there was no significant color change in all the treatments (**b**).

In the Hass cultivar, the plastic film treatment resulted in full color retention, since after 21 days in the storage chamber, the avocados retained almost the same color as the beginning of the experiment. This means that color-wise, the avocados after 21 days were not discolored, as is typically the case during ripening of the Hass cultivar. Additionally, it is also observed that in the Ethrel treatment and in the control, there is a decrease of −30% in the L parameter and an increase of 110% in parameter *a* of the color. This phenomenon may be attributed to the destruction of anthocyanins, which results in a change in color (Figure 1a). The Fuerte cultivar typically does not exhibit any discoloration during the ripening process.

In contrast, in the Fuerte cultivar, the result was not so different, since the only significant difference was limited to the color parameter a, which shows the variation of shades from green to black, which occurs in avocados during ripening, resulting in the shades decreasing over time (Figure 1b).

On the results of color measurements, ANOVA analysis was performed. The *p* value for the Hass cultivar is 1.04086 × 10^−9^ and that for Fuerte cultivar is 3.73064 × 10^−14^. Both are below 5%, indicating they are statistically different. Also, Tukey’s HSD post hoc test was performed to determine which groups are significantly different. The results are indicated with capital letters in parentheses on the graph.

### 3.2. Titratable Acidity Results

The results of the titratable acidity measurements are expressed in mL of NaOH (0.01 M) consumed until a permanent color change is observed. In Table 1, in the plastic film treatment, the Hass cultivar compared to the control shows an increase of about 0.5 mL of NaOH (0.01 M), while in the treatment with Ethrel, the difference with the plastic film treatment is increased by about 0.8 mL of NaOH (0.01 M), and in relation to the control, there is an increase of 1.4 mL of NaOH (0.01 M).

In the titratable acidity results presented in Table 1, there is a difference in the behavior between the Hass and Fuerte cultivars. In the Hass cultivar without any treatment (21 d storage untreated), the titratable acidity was almost the same as that of the original control (4% drop). In contrast, in the Ethrel treatment, there is a large increase in titratable acidity of 76%. In the treatment with the plastic film, there is an increase in titratable acidity of 27%. We observe that in the Hass cultivar, the plastic film treatment had a better effect on the titratable acidity than the Ethrel treatment, but not as good as without any treatment (control).

On the results of TA, ANOVA analysis was performed. The *p* value for the Hass cultivar is 0.00878 and that for the Fuerte cultivar is 0.019. Both are below 5%, indicating they are statistically different. Also, Tukey’s HSD post hoc test was performed to determine which groups are significantly different. The results are indicated with letters in the table.

### 3.3. Total Soluble Solids Results

Table 2 shows that the Hass cultivar had the highest percentage of total soluble solids in the plastic film treatment, compared to Fuerte. Furthermore, the Hass cultivar in the plastic film treatment shows twice the difference compared to the control and one Brix unit compared to the Ethrel treatment. In contrast, the plastic film treatment had the lowest percentage of total soluble solids in the Fuerte cultivar, showing the reverse effect from Hass.

In the results of total soluble solids in the Fuerte cultivar, there is an increase in Brix value of 55% in the untreated treatment. In the plastic film treatment, there is an increase of 22% and an increase of 66% in the Ethrel treatment. From these results, it can be observed that the Fuerte cultivar shows the greatest increase in total soluble solids in the Ethrel treatment, while the plastic film treatment shows the smallest increase in total soluble solids. This result means that the plastic film treatment has delayed the ripening of the fruit.

The total soluble solids results for the Hass cultivar show the opposite result compared to Fuerte. In the case without treatment, there was a slight increase of 6%. In the plastic film treatment, there was a significant increase in the Brix value of 120%, while in the Ethel treatment, there was an increase of 53%. The plastic film treatment showed the greatest increase in total soluble solids for the Hass cultivar.

### 3.4. Flesh Consistency Results

Table 3 shows the results for flesh consistency. Without treatment, the consistency of the Fuerte cultivar was reduced by 88%, while the Ethrel treatment resulted in a reduction of 92%. The plastic film treatment led to a reduction of only 13%, leading to significantly harder fruit. In the Hass cultivar, consistency without treatment was reduced by 83%, while that in the Ethrel treatment was reduced by 93%, while in the plastic film treatment, the reduction was only 5.5%.

On the results of flesh consistency, ANOVA analysis was performed. The *p* value for the Hass cultivar is 1.61904 × 10^−15^ and that for the Fuerte cultivar is 4.60287 × 10^−20^. Both are below 5%, indicating they are statistically different. Also, Tukey’s HSD post hoc test was performed to determine which groups are significantly different. The results are indicated with letters in the table.

### 3.5. Respiration Results

Table 4 shows that both cultivars exhibit similar respiration behavior. The CO_2_% release rate without treatment in Fuerte was increased by 165% compared to the beginning of the experiment. In the plastic film treatment, the increase was higher at 210%, and the Ethrel treatment had the smallest increase of 62%. The results for the Hass cultivar showed a similar behavior. Without treatment, the increase was 71%; in the plastic film treatment, the increase was slightly greater at 75%; and in the Ethrel treatment, the increase was lower at 22%. The plastic film treatment had the highest increase in the CO_2_% release rate, while the treatment with Ethrel had the lowest.

On the results of flesh consistency, ANOVA analysis was performed. The *p* value for the Hass cultivar is 0.479347399 and that for the Fuerte cultivar is 0.009940008. Also, Tukey’s HSD post hoc test was performed to determine which groups are significantly different. The results are indicated with letters in the table.

### 3.6. PAL Activity Results

**Figure 2:**
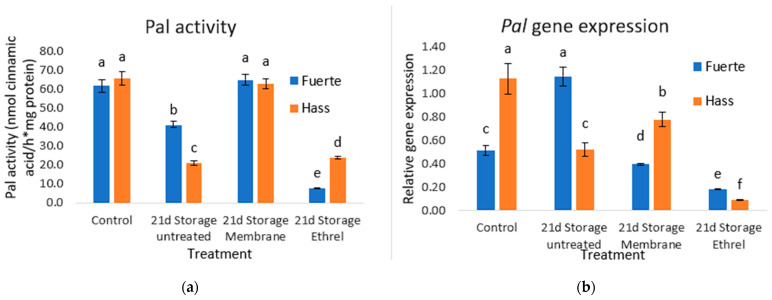
(**a**) PAL activity is expressed in nmol cinnamic acid/h*mg protein. In plastic film treatment, PAL activity was maintained at levels comparable to the control. (**b**) PAL gene expression generally follows the activity model except for the Fuerte fruit stored for 21 days without treatment. Different small letters indicate statistically significant changes, as compared to the Hass control (post hoc analyses).

The results of the PAL activity measurement indicate that the plastic film treatment is effective in both cultivars of the experiment. More specifically, in the Fuerte cultivar without treatment, stored for 21 days, a reduction of 33.9% is observed. On the contrary, the plastic film treatment shows a slight increase of 4.8%, and, finally, the treatment with Ethrel shows a large reduction of 87%. The Hass cultivar without treatment, stored for 21 days, shows a large reduction of 68.2%. The plastic film treatment shows a very small decrease of 4.5% and the Ethrel treatment shows a large decrease of 63.6%. PAL activity and PAL gene expression can be seen in Figure 2.

### 3.7. Total Phenolic Content

**Figure 3:**
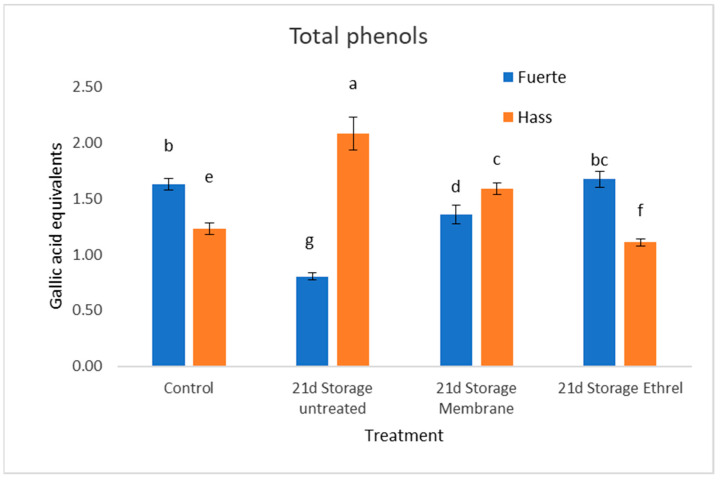
Total phenolic contents (mg/g FW) expressed as gallic acid equivalents. The Fuerte cultivar shows a decrease without treatment, followed by an increasing trend with the treatments. In contrast, the Hass cultivar exhibits an increase without treatment, followed by a downward trend with the treatments. Different small letters indicate statistically significant changes, as compared to the Hass control (post hoc analyses).

Significant differences in total phenolic measurements were observed between the Hass and Fuerte cultivars. In the case of Fuerte without treatment (21 d storage untreated), there is a reduction of 50.6%. In the case of the plastic film treatment, there is a slight decrease of 16%, and, finally, in the case of the Ethrel treatment, there is a slight increase of 3%. In the Hass cultivar, the results are very different, since in the case without treatment (21 d storage untreated), there is a significant increase of 69%; in the case with the plastic film treatment, there is an increase of 29%; and, finally, in the case with Ethrel, there is a decrease of 9.7%. The results can be seen in Figure 3.

### 3.8. APX Activity Results

**Figure 4:**
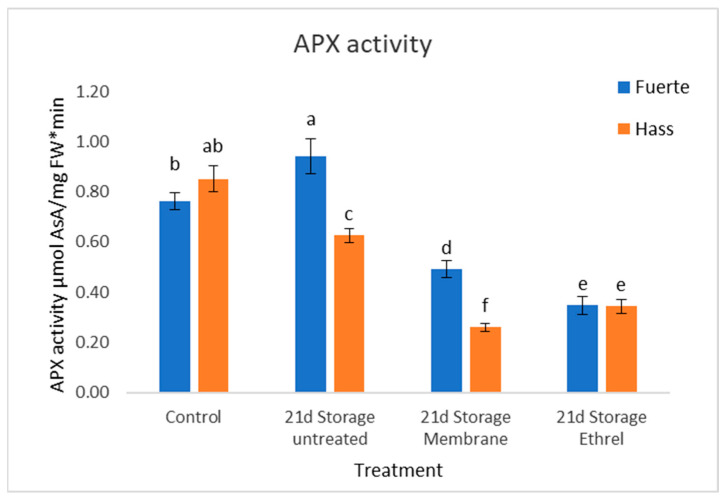
APX activity results (μmol AsA/mg FW*min). There are slight differences in the behavior between the two cultivars in the experiment. In the Hass cultivar, the plastic film treatment seems to have the greatest reduction compared to Fuerte. Different small letters indicate statistically significant changes, as compared to the Hass control (post hoc analyses).

The activity of ascorbate peroxidase (APX) showed minor differences in behavior between the two experimental cultivars. In the Fuerte cultivar without treatment stored for 21 days, there is an increase of 23%. In the treatment with the plastic film, there is a decrease of 35.6%, and in the treatment with Ethrel, there is a decrease of 54.4%. In the Hass cultivar without treatment stored for 21 days, there is a reduction of 26.4%. The plastic film treatment results in the greatest decrease of 69.3% and the Ethrel treatment shows a decrease of 59.5%. Notably, the plastic film treatment has the greatest reduction in the Hass cultivar. The APX results can be seen in Figure 4.

### 3.9. β-Gal Activity Results

**Figure 5:**
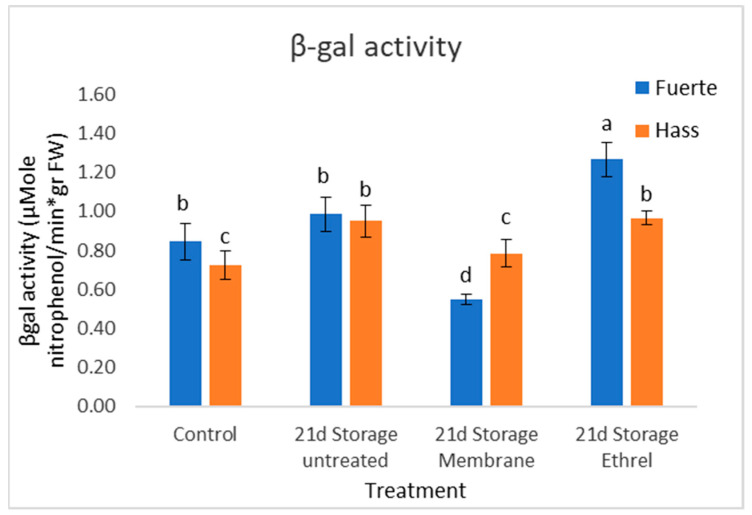
β-gal activity (μMole nitrophenol/min*gr FW). The β-galactosidase activity was higher in fruits stored after Ethrel treatment and in fruits stored without any treatment. However, in fruits stored using plastic film, the enzyme activity was significantly lower (Fuerte cultivar) or comparable to freshly harvested fruits. Different small letters indicate statistically significant changes, as compared to the Hass control (post hoc analyses).

**Figure 6:**
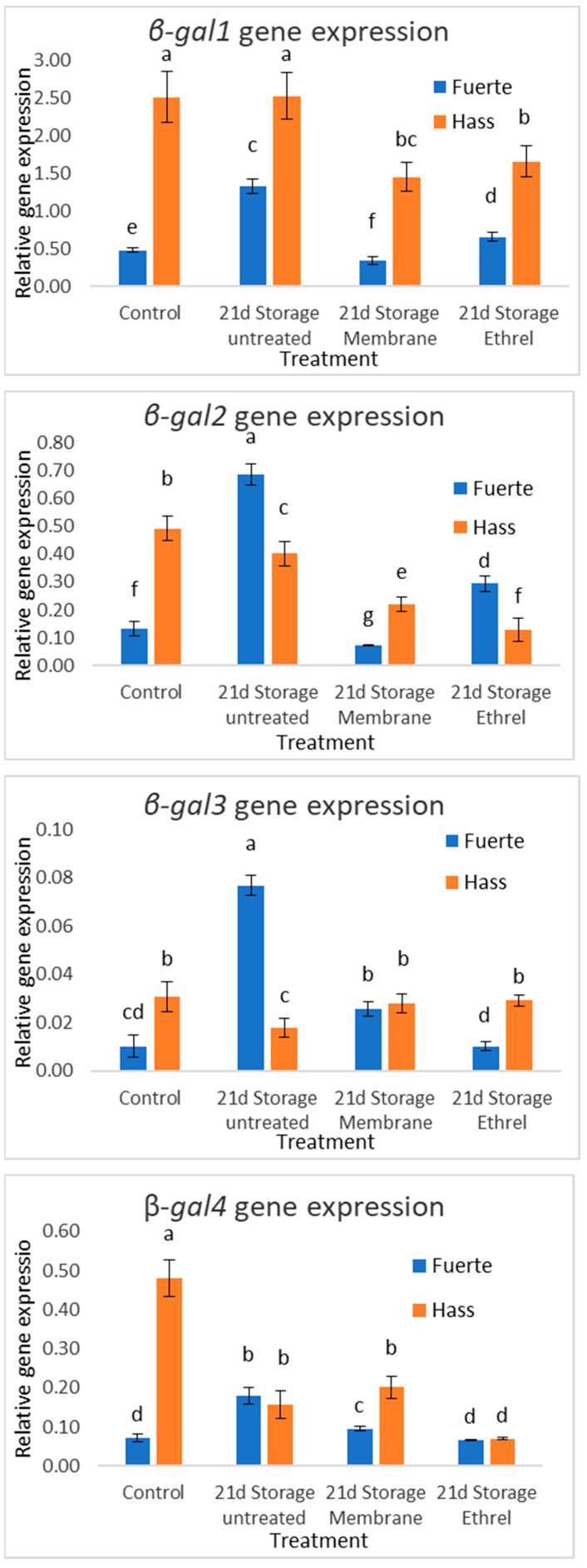
Expression of β-gal genes 1–4. The experimental treatments resulted in significant differences in the expression patterns of genes encoding β-galactosidase isoenzymes. However, β-gal1 exhibited a comparable expression pattern to the enzyme activity in the Hass cultivar, as did β-gal2 in the Fuerte cultivar. β-gal3 exhibited a different pattern with respect to enzyme activity for both cultivars. Different small letters indicate statistically significant changes, as compared to the Hass control (post hoc analyses).

Previous scientific studies have emphasized the significance of β-galactosidase in the softening of avocado fruit [20] as the enzyme is considered a dominant regulator of the modification of fruit cell membranes that occurs during ripening. The activity of β-galactosidase was higher in fruits stored after Ethrel treatment, as well as in fruits stored without any treatment. In fruits stored using plastic film, the enzyme activity was significantly lower (Fuerte cultivar) or comparable to freshly harvested fruits. Expression patterns of genes encoding β-galactosidase isoenzymes showed significant differences between treatments in the experiment. However, β-galactosidase 1 (β-gal1) showed an expression pattern comparable to the enzyme activity as did β-gal2 with respect to the Fuerte cultivar. β-gal3 showed a different pattern with respect to the enzyme activity for both cultivars. Enzymes that cause fruit softening (mainly hydrolases) such as β-galactosidase are inhibited by the use of treatments that delay the ripening process such as the use of plastic film and 1-MCP [20]. As a result, increased cohesiveness and reduced soluble solids concentration are observed. Figure 5 and Figure 6 show the results of β-gal activity and gene expression.

The β-galactosidase measurements showed similar results in both cultivars. Without treatment, the Fuerte cultivar had a slight increase of 16.5%. However, with the plastic film treatment, there was a decrease of 35%, while with the Ethrel treatment, there was a large increase of 49.7%. Without treatment, the Hass cultivar had an increase of 31%. The results indicate that the plastic film treatment is more effective, with a slight increase of 8.2%, compared to the Ethel treatment which showed an increase of 33.1%. These results show that the plastic film treatment shows the best results since it has the lowest value. β-galactosidase is an enzyme that is associated with the softening of fruit tissues. Therefore, the higher the rate of its increase, the higher the softening of the fruit.

### 3.10. qPCR Results

Table 5 shows the gene primer sequences that were used for qPCR for six genes *β-Gal 1, β-Gal 2, β-Gal 3, β-Gal 4, Pal, Actin*.

## 4. Discussion

The two cultivars, Hass and Fuerte, were specifically chosen for inclusion in the current study because they are highly prevalent in the region of Crete. In particular, within the area of Chania, these two cultivars dominate the landscape of avocado cultivation, representing the majority of the total avocado production. This made them readily available for our study.

Regarding the macroscopic appearance of the fruit (Figure 1), the flesh of Hass avocados treated with Ethrel exhibited a soft, buttery texture and a blackened skin. In the case of the Fuerte cultivar, the skin remained relatively green when treated with Ethrel. With the plastic film treatment, there was little difference in the appearance of the flesh for both cultivars. Without treatment, both cultivars exhibited normal ripening. In his study, Hertog [21] studied the ripening of avocado as the color changes from green to black and similar color changes were observed in the results of the current study.

The titratable acidity results presented in Table 1 show a different behavior between the Hass and Fuerte cultivars. In the Hass cultivar, without any treatment, the titratable acidity was almost the same as in the initial control (4% decrease). The Ethrel treatment, on the other hand, showed a strong increase in acidity of 76%. In the plastic film treatment, there was a 27% increase in acidity. It can be seen that in the Hass cultivar, the plastic film treatment had a better effect on acidity than the Ethrel treatment. However, without treatment, the resulting acidity was lower. In the current research, Ethrel caused a significant increase in titratable acidity of the cultivar Hass. Previous research results [22] have shown that the red ripe tomato fruits “Roma VF” stored at 5 °C with exogenous ethylene exhibit superior appearance and higher respiration and senescent rates, as compared to the control. On the contrary, the green mature fruits presented an opposite effect when treated with ethylene, possibly due to an auto-induction mechanism in the synthesis process of the enzymes that are involved in ethylene biosynthesis controlled by the phytohormone ethylene [23]. Thus, green mature fruits, when exposed to C_2_H_4_, revealed a negative feedback mechanism.

In the case of the Fuerte cultivar, the opposite effect was observed. In other words, without any treatment, the titratable acidity increased by 41%. In the case of the plastic film treatment, there is a decrease of 23%, while the Ethrel treatment shows a decrease of 17%. In the case of the Fuerte cultivar, the treatment with plastic film shows the best results as it displayed the lowest acidity levels.

The results of the total soluble solids (Table 2) in Fuerte show a 55% increase in Brix value in the untreated measurement. The plastic film treatment resulted in a 22% increase, while the Ethrel treatment resulted in a 66% increase. These results indicate that the Ethrel treatment had the greatest increase in total soluble solids for the Fuerte cultivar, while the plastic film treatment had the smallest increase. It can be concluded that the plastic film treatment delayed the ripening of the fruit [7,24].

The results for total soluble solids for the Hass cultivar show the opposite result to Fuerte. In the case without treatment, there was a slight increase of 6%. In the plastic film treatment, there was a large increase in Brix value of 120%, while in the Ethel treatment, there was an increase of 53%. In the Hass cultivar, the plastic film treatment showed the greatest increase in total soluble solids.

In the measurement of flesh consistency results (Table 3), the plastic film treatment resulted in the firmest fruits in both cultivars of the experiment. In the untreated measurement, the Fuerte cultivar had an 88% reduction in firmness and the Ethrel treatment had a 92% reduction. The plastic film treatment had a reduction of only 13%, resulting in much harder fruit. For the Hass cultivar, consistency was reduced by 83% without treatment and by 93% with Ethrel. With the plastic film treatment, the reduction was only 5.5%.

The results of the respiration measurements show a similar behavior between the two cultivars. In the Fuerte cultivar, the release rate of CO_2_% without treatment increased by 165% compared to the beginning of the experiment. The plastic film treatment resulted in a higher increase of 210%, while the Ethrel treatment had the smallest increase of 62%. The results for the Hass cultivar showed a similar behavior. The plastic film treatment resulted in the highest increase in CO_2_% release rate, while the Ethrel treatment resulted in the lowest. The untreated sample showed a 71% increase in CO_2_% release rate. The sample treated with plastic film showed a slightly greater increase of 75%, while the sample treated with Ethrel showed a decrease of 22%. The plastic film treatment resulted in the highest increase in CO_2_% release rate, while the Ethrel treatment resulted in the lowest.

Significant differences were found in total phenolic contents (Figure 3) between the Hass and Fuerte cultivars. In Fuerte, there was a decrease in total phenolic contents without any treatment, followed by an increase with the treatments. Conversely, in the Hass cultivar, there was an increase in total phenolic measurements without treatment, followed by a downward trend with the treatments.

Polyphenols, a significant secondary metabolite in avocado, show a key role in protection from (a)biotic stress and assist as effective antioxidant compounds once eaten by people. PAL is the central enzyme regulator, opening a biochemical pathway to polyphenol production. The use of plastic film during storage had the effect of maintaining the activity of the enzyme at levels comparable to the control. The PAL activity in stored avocados that were not treated was higher than in fruits that were treated with Ethrel. Gene expression as a model generally follows the activity except for the Fuerte fruit stored for 21 days without treatment. Postharvest treatments on avocado fruits that prolong the postharvest life of fruits are associated with enhanced PAL activity. The production of secondary metabolites from the phenolic pathway is associated, among others, with the resistance to postharvest rot [25] and with the higher antioxidant activity of fruits [26]. The PAL activity and PAL gene expression can be seen in Figure 2.

The activity of ascorbate peroxidase (APX) showed minor differences in behavior between the two experimental cultivars. It is also interesting that in the Ethrel treatment, the two cultivars had the same percentage. During the process of measuring APX in the Fuerte cultivar, a little browning was observed in the tissue. The measurement did not show stabilization at any value; therefore, an evaluation of the results of the action was made. It was also necessary to use a filter for acids; this way, the values would probably be clearer while still retaining the reliability of the measurement. The APX results can be seen in Figure 4.

The modification of the cell walls represents the central issue affecting avocado softening. β-galactosidase (β-Gal), one of the cell-wall-modifying enzymes, plays a vital role in avocado ripening/softening. The results of β-gal activity shown in Figure 5 and Figure 6 lead to the conclusion that the use of plastic film resulted in an effective reduction in the activity of β-galactosidase, as shown by the results of measuring the rate of the hydrolysis reaction (enzyme activity) and the expression of the genes. β-galactosidase is encoded by a large family of enzymes with different regulations as they are involved in a wide cultivar of physiological processes. This probably accounts for the differences in gene expression patterns. This fact was also recorded by [27] who reported that the β-gal1 isoenzyme is the one associated with fruit ripening and softening, while the other isoenzymes are probably involved to some extent with galactose metabolism in cells and cell walls. Finally, the reduced relative expression of the genes encoding β-galactosidase in fruits treated with Ethrel is probably due to the fact that in these fruits, over-ripening has started and there is probably now a reduced availability of substrates leading to a reduction in gene expression. This phenomenon has been observed in over-ripe tomato fruits [28].

## 5. Conclusions

The treatment of avocados with a plastic film cover (membrane) has shown excellent results in retaining quality characteristics and enzyme activities via maintaining firmness in higher levels. This treatment has the potential to extend the postharvest life of avocados without the use of chemicals.

The treatment with Ethrel had the effect of increasing the Brix value in the Fuerte cultivar, resulting in fruit that is “sweeter” in taste, which is a positive quality characteristic. In both cultivars, the fruits treated with Ethrel exhibited uniform ripening compared to those without treatment. In the total phenolics measurements, Ethrel treatment resulted in an increase in the Fuerte cultivar.

Our study showed that the plastic film treatment had a positive effect on PAL activity in both cultivars as the activity of PAL was maintained at high levels comparable to the control. Postharvest interventions that extend the postharvest life of avocado fruit are linked to increased PAL activity.

β-galactosidase is considered a dominant regulator for the modification of fruit cell membranes that occurs during ripening [21]. When fruits were stored using a plastic film, the activity of the enzyme was significantly lower (Fuerte) and comparable to freshly harvested fruits. The observations made in the experiment lead to the conclusion that the use of plastic film resulted in an effective reduction in the activity of β-galactosidase, which is an enzyme that is associated with the softening of fruit tissue. Therefore, the higher the rate of its increase, the softer the fruit becomes.

The results are quite interesting, but further research on other cultivars is needed in order to draw broader conclusions. In addition, further research should be conducted to investigate other parameters that were not examined in the current experiment. It is also important to investigate other methods of modified atmospheres in avocados, for example, individual packaging. Elucidation of the candidate genes encoding key enzymes in the affected metabolic pathways will further aim to facilitate the exploration of related gene networks and eventually lead to systems biology approaches in avocado fruit quality.

## Figures and Tables

**Table 1:** Titratable acidity results expressed in mL of NaOH (0.01 M) for the treatments in both cultivars (1 mL of 0.01 M NaOH equals 0.01 milliequivalents of Na). In the Hass cultivar, control–Ethrel and 21 days storage untreated–Ethrel, we found a statistical difference in Tukey’s HSD post hoc pairwise analysis. Different small letters (in parentheses, next to average values) indicate statistically significant changes, as compared to the control.

Titratable Acidity—mL of NaOH (0.01 M)				
	Control	21 Days Storage Untreated	Plastic Film	Ethrel
Hass	1.80 (a)	1.73 (a)	2.28 (a)	3.17 (b)
SD	0	0.21	0.63	0.48
Fuerte	1.60 (a)	2.27 (b)	1.23 (a)	1.33 (b)
SD	0.23	0.25	0.55	0.12

**Table 2:** Total soluble solids results showing Brix value and pH for the treatments in both cultivars. ± indicates SD values. Different small letters (in parentheses, next to values) indicate statistically significant changes, as compared to the control.

	Total Soluble Solids (Brix Value)				
	Fuerte			Hass	
	pH	Brix Value		pH	Brix Value
Control	6.070 ±0.031 (a)	0.9 ± 0.07 (a)	Control	6.100 ± 0.009 (a)	1.5 ± 0.05 (a)
Untreated *	6.160 ± 0.015 (b)	1.4 ± 0.13 (b)	Untreated *	6.220 ± 0.011 (b)	1.6 ± 0.06 (a)
Plastic film	6.425 ± 0.017 (c)	1.1 ± 0.11 (c)	Plastic film	6.330 ± 0.012 (c)	3.3 ± 0.07 (b)
Ethrel	6.372 ± 0.016 (d)	1.5 ± 0.14 (b)	Ethrel	6.235 ± 0.005 (d)	2.3 ± 0.09 (c)

Untreated * = 21 days storage untreated.

**Table 3:** Flesh consistency results expressed in Kg/cm^2^ for the treatments in both cultivars. Different small letters (next to average values) indicate statistically significant changes, as compared to the control.

**Fuerte Flesh Consistency Kg/cm^2^**				
	Control	21 d storage Untreated	Plastic film	Ethrel
Average	18.91875 (a)	2.2875 (b)	16.45625 (c)	1.4125 (b)
SD	0.40	1.52	2.91	0.58
**Hass Flesh Consistency Kg/cm^2^**				
	Control	21 d storage Untreated	Plastic film	Ethrel
Average	17.75625 (a)	2.9625 (b)	16.775 (a)	1.06875 (b)
SD	0.04	3.49	3.28	0.40

**Table 4:** Respiration rate results expressed in mL CO_2_ kg^−1^ h^−1^ for the treatments in both cultivars. Different small letters (next to average values) indicate statistically significant changes, as compared to the control.

Respiration Rate of Fuerte (mL CO_2_ kg^−1^ h^−1^)				Respiration Rate of Hass (mL CO_2_ kg^−1^ h^−1^)			
	Without Treatment	Plastic Film	Ethrel		Without Treatment	Plastic Film	Ethrel
Average	15.11125 (a)	13.95142857 (a)	8.94125 (b)	Average	16.4225 (a)	15.455 (a)	11.955 (a)
SD	3.76	2.47	4.71	SD	8.39	6.05	8.19

**Table 5:** Gene primer sequences used for qPCR.

Gene	Encoded Enzyme/Protein	Primer Sequence	Accession Number
*β-Gal 1*	Beta galactosidase 1	Sense: GTGGGAGATAGGTGCCATCG	AB061017
		Antisense: CCAGCCAGTCCAGAGTTCAG	
*β-Gal 2*	Beta galactosidase 2	Sense: GGCGAGTGAGGTTTCTCCAA	AB252827
		Antisense: AAGCCTGCCCCATCTTTCTC	
*β-Gal 3*	Beta galactosidase 3	Sense: GGACTTCCTGGTTTACGGCT	AB252828
		Antisense: ATGTGACTCCTGGGAACTGC	
*β-Gal 4*	Beta galactosidase 4	Sense: GCATTTGCGTTGTGCAATGG	AB252829
		Antisense: AAGCTCCCACAAGTTCCCAG	
*Pal*	Phenylalanine ammonia lyase	Sense: CAATGGAGGATCCGGCCACA	U16130
		Antisense: GTGGCAAACATGGGGTGATG	
*Actin*	Actin	Sense: GTTATGGTTGGGATGGGGCA	GU272027
		Antisense: TCCCTGTTGGCTTTTGGGTT	

## Data Availability

Data are contained within the article. The original contributions presented in this study are included in the article. further inquiries can be directed to the corresponding author.
